# Persistence of fatigue in the absence of pathophysiological mechanisms in some patients more than 2 years after the original SARS‐CoV‐2 infection

**DOI:** 10.1113/EP092850

**Published:** 2025-07-20

**Authors:** Giovanni Baldassarre, Lucrezia Zuccarelli, Thomas Favaretto, Caterina Ursella, Andrea Palomba, Paulo Cesar do Nascimento Salvador, Emanuela Sozio, Ernesto Crisafulli, Massimo Imazio, Carlo Tascini, Bruno Grassi

**Affiliations:** ^1^ Department of Medicine (DMED) University of Udine Udine Italy; ^2^ Division of Infectious Diseases Azienda Sanitaria Universitaria Friuli Centrale (ASUFC) Udine Italy; ^3^ Department of Medicine University of Verona Verona Italy; ^4^ Cardiothoracic Department Azienda Sanitaria Universitaria Friuli Centrale (ASUFC) Udine Italy

**Keywords:** exercise intolerance, exercise ventilatory inefficiency, long COVID, oxidative metabolism

## Abstract

Following an acute infection with severe acute respiratory syndrome coronavirus 2 (SARS‐CoV‐2), a substantial percentage of patients report the persistence of debilitating symptoms, often grouped in a syndrome termed ‘long COVID’. We sought to identify potential pathophysiological mechanisms responsible for the persistence, in some long COVID patients, of symptoms related to fatigue/exercise intolerance (excessive or early fatigue, excessive or early dyspnoea, muscle weakness, and myalgias) more than 2 years after the original infection. Twelve patients who reported persistent symptoms (Long COVID group; 57 ± 6 years, mean ± SD), and 14 patients without the symptoms (Control group; 57 ± 8 years) were evaluated. An extensive series of measurements were performed to identify pathophysiological mechanisms potentially responsible for the symptoms. In long COVID patients, all items evaluating quality of life (SF‐36 questionnaire) had lower scores (*P *< 0.01) compared to control. The habitual level of physical activity, muscle size and strength, maximal aerobic power and the ventilatory thresholds, peak cardiac function, the mechanical efficiency of cycling, pulmonary ${\dot{V}}_{O_{2}}$ kinetics, microvascular/endothelial function (hyperemic response in the common femoral artery during passive leg movements), skeletal muscle oxidative metabolism (peak fractional O_2_ extraction and muscle ${\dot{V}}_{O_{2}}$ recovery kinetics by the repeated occlusions test, by near‐infrared spectroscopy) were not different in the two groups. Evidence of ventilatory inefficiency was described in a subgroup of long COVID patients. More than 2 years after the original SARS‐CoV‐2 infection, a discrepancy was observed between the persistence of debilitating symptoms of fatigue/exercise intolerance and the absence of several investigated pathophysiological mechanisms. The discrepancy may be due to factors that remain to be elucidated.

## Abstract (highlights)

- **What is the central question of this study?**
  Which pathophysiological mechanisms are responsible for the persistence, in some patients, seen more than 2 years after the original SARS‐CoV‐2 infection, of symptoms of fatigue/exercise intolerance?
- **What is the main finding and its importance?**
  A discrepancy was observed between the persistence of symptoms of fatigue/exercise intolerance (excessive or early fatigue, excessive or early dyspnoea on exertion, muscle weakness, and myalgias) and the absence of several investigated pathophysiological mechanisms. Symptoms may be attributable to other factors (i.e., psychological/neurological) that were not investigated.

## 1 INTRODUCTION

Following an acute infection with severe acute respiratory syndrome coronavirus 2 (SARS‐CoV‐2) and the ensuing disease (coronavirus disease 2019, or COVID‐19), which reached pandemic dimensions and affected millions of people worldwide, a substantial percentage of patients reported persisting and often debilitating symptoms, lasting for several months following the recovery from the acute disease (Davis et al., 2023). These symptoms, which may affect multiple organ systems (Davis et al., 2023), are often grouped in a syndrome termed ‘long COVID’ or ‘post‐acute sequelae of COVID‐19’ (PASC) (Davis et al., 2023). Among the symptoms, early or excessive fatigue is one of the most prevalent (Nasserie et al., 2021; Serviente et al., 2022; Soares et al., 2022), up to 2 years after acute infection, regardless of initial disease severity (Huang et al., 2022). Although difficult to define (Kluger et al., 2013), a consensus has been recently reached in defining fatigue as a ‘range of symptoms from mild subjective feelings of tiredness to an overwhelming, debilitating and sustained sense of exhaustion that likely decreases one's ability to execute daily activities and function normally in familiar or social roles (Maxwell et al., 2024). This definition is akin to the definition of ‘perceptual fatigue’ in the study by Kluger et al. (2013). In association with other exercise intolerance‐related symptoms belonging to the long COVID syndrome, such as excessive or early dyspnoea on exertion, muscle weakness and myalgias, fatigue plays a significant role in limiting the patients’ quality of life (Kluger et al., 2013; Ma et al., 2022).

Numerous studies have been conducted in recent years, mostly after a few weeks or months following an acute COVID‐19 illness, in order to investigate the pathophysiological mechanisms responsible for the signs and symptoms of the long COVID syndrome. These pathophysiological mechanisms included microvascular/endothelial dysfunction (Paneroni et al., 2021; Ratchford et al., 2021; Serviente et al., 2022), possibly associated with a persistent low‐level inflammation and increased oxidative stress; skeletal muscle dysfunction, associated with impairments of mitochondrial respiratory function and oxidative metabolism (Appelman et al., 2024; Baratto et al., 2021; Colosio et al., 2023; Jamieson et al., 2024; Singh et al., 2022); decreased mechanical efficiency (Pleguezuelos et al., 2021); slower pulmonary O_2_ uptake (${\dot{V}}_{O_{2}}$) on‐kinetics and greater O_2_ deficit (Colosio et al., 2023; Longobardi et al., 2022); reduced muscle mass and strength (Ramírez‐Vélez et al., 2023); and inactivity and muscle deconditioning (Rinaldo et al., 2021). The persistence of symptoms of fatigue/exercise intolerance and their relationship with pathophysiological mechanisms, after longer periods following the original infection, is of interest, particularly after considering that the patients, following the initial pandemic of COVID‐19, likely underwent multiple vaccinations and further infections with different strains of the virus. Indeed, as recently reported by Xie et al. (2024), although the cumulative incidence of long COVID during the first year after SARS‐CoV‐2 infection decreased over the course of the pandemic, the risk of long COVID seems to be substantial even among vaccinated persons who had SARS‐CoV‐2 infection in the ‘omicron era’ (the most recent era of the pandemic).

Within this perspective, the aim of the present study was to investigate several pathophysiological mechanisms potentially responsible for the persistence of fatigue and other exercise intolerance‐related symptoms (early or excessive dyspnoea on exertion, muscle weakness, myalgias), more than 2 years after an original infection with the initial strains of SARS‐CoV‐2. To address this question an extensive series of non‐invasive measurements were conducted evaluating cardiorespiratory, microvascular/endothelial and skeletal muscle functions in patients, tested more than 2 years after an original SARS‐CoV‐2 infection, who presented (‘Long COVID’ group) or did not present (‘Control’ group) the symptoms mentioned above. The aim was to establish a link between the symptoms and the investigated pathophysiological mechanisms. We hypothesized the presence of one or more of the investigated pathophysiological mechanisms in the participants with fatigue/exercise intolerance symptoms, and the absence of the pathophysiological mechanisms in the participants without the symptoms.

## 2 METHODS

### 2.1 Ethical approval

The study protocol was approved by the Ethical Committee of the Friuli Venezia Giulia Region (CEUR‐2020‐OS‐219/CEUR‐2020‐OS‐205). All procedures conformed to the standards set by the *Declaration of Helsinki*. All participants gave their written informed consent after they received a detailed explanation of the experimental procedures before the start of the study.

### 2.2 Participants

Twenty‐six patients of both sexes were recruited approximately 2 years after being evaluated at the Division of Infectious Diseases, Azienda Sanitaria Universitaria Friuli Centrale (ASUFC), Udine, Italy, for a SARS‐CoV‐2 infection occurring between March and April 2020 and enrolled in the CORMOR study (Peghin et al., 2021). Among these patients, 12 complained of persistence of excessive or early fatigue and other debilitating symptoms of exercise intolerance referable to the long COVID syndrome (Soriano et al., 2022) such as excessive or early dyspnoea on exertion, muscle weakness, and myalgias (‘Long COVID’ group). Fourteen participants (‘Control’ group), matched by age, sex and body mass index (BMI), who did not report any of the symptoms mentioned above after the initial SARS‐CoV‐2 infection, were also enrolled.

General characteristics, associated conditions and the main utilized drugs in the patients of the two study groups are summarized in Table 1.

**TABLE 1:** General characteristics, comorbidities and medications of control participants and of patients with some persistent long COVID symptoms.

	Control (*n* = 14)	Long COVID (*n* = 12)	*P*
Male sex (*n*)	8	6	
Age (years)	57 ± 8	57 ± 6	0.78
Height (m)	1.71 ± 0.08	1.74 ± 0.10	0.39
BM (kg)	73.7 ± 14.6	77.9 ± 10.5	0.42
BMI (kg m^−2^)	25.0 ± 3.6	25.7 ± 2.2	0.61
FFM (% BM)	77.2 ± 6.8	75.3 ± 4.1	0.46
FM (% BM)	22.6 ± 6.7	24.8 ± 4.2	0.38
Thigh volume (mL)	5450 ± 1176	5443 ± 1400	0.99
Quadriceps muscle mass (kg)	2.02 ± 0.43	2.02 ± 0.36	0.99
Long COVID symptoms			
Fatigue (*n*)	0	12	
Dyspnoea (*n*)	0	6	
Myalgias (*n*)	0	5	
Muscle weakness (*n*)	0	5	
Time elapsed between initial SARS‐CoV‐2 infection and assessment (months)	25 ± 4	27 ± 6	0.34
Variables related to COVID‐19 hospitalization			
Hospitalization (*n*)	4	3	
Need of ventilatory support (*n*)	1	0	
Need of oxygen therapy (*n*)	0	2	
ICU admission (*n*)	3	0	
Comorbidities			
Hypertension (*n*)	4	3	
Hypercholesterolemia (*n*)	3	5	
Osteoporosis (*n*)	1	0	
Hypothyroidism (*n*)	1	1	
Medications			
Antihypertensives (*n*)	4	3	
Anticoagulants and antiplatelets (*n*)	1	1	
Diuretics (*n*)	0	1	
Lipid‐lowering agents (*n*)	1	2	

*Note*: Data are means ± SD. Abbreviations: BM, body mass; BMI, body mass index; FFM, fat free mass; FM, fat mass; ICU, intensive care unit; SARS‐CoV‐2, severe acute respiratory syndrome coronavirus 2.

At the time of the assessment, the recruited patients were no longer positive for the SARS‐CoV‐2 infection and were in stable clinical conditions. All participants were fully vaccinated (≥2 doses), and before being admitted to the trial underwent a medical screening. Participants with neurological, cardiovascular, respiratory or orthopaedic diseases contraindicating or preventing the execution of a cycle ergometer test were excluded from the study. All participants were sedentary or moderately active before the infection; individuals participating in sport activities or physically very active were excluded from the study. Also, the patients refusing to undergo an incremental exercise test on a cycle ergometer were excluded from the study.

### 2.3 Experimental protocol

All participants visited the laboratory once, and the experimental session lasted ∼2.5 h. Participants arrived at the laboratory in the early afternoon, at least 2 h postprandial, and were instructed to avoid caffeine and alcohol on the day of the visit, and also to abstain from strenuous physical activity for at least 24 h prior to the testing session. Measurements were performed in the order of presentation of the ‘Measurements’ section below. All measurements were conducted in a dedicated laboratory located at the Division of Infectious Diseases, ASUFC, and standard safety procedures against the risk of transmission of COVID‐19 infection were followed. Tests were conducted under continuous medical supervision. Before data collection, participants were allowed enough time to become familiar with the researchers and with the set‐up environment, as well as with the experimental protocol, by performing short preliminary practice runs. All the proposed measurements were non‐invasive and were separated by adequate recovery periods.

### 2.4 Measurements

#### 2.4.1 Questionnaires

Physical activity in the period in which the tests were performed was assessed by the International Physical Activity Questionnaire Short Form (IPAQ‐SF; Lee et al., 2011) and reported as metabolic equivalent‐minutes per week (MET min week^−1^).

The MOS 36‐Item Short‐Form Health Survey (SF‐36) was administered in order to evaluate the patients’ functional status, well‐being and other health care outcomes. Scores for each domain were transformed to a 0–100% scale, with a higher score defining a more favourable health state.

#### 2.4.2 Anthropometric measurements

Leg circumferences and heights, together with skinfold measurements, were collected to estimate thigh volume (Jones & Pearson, 1969) and to calculate quadriceps femoris muscle mass (Andersen & Saltin, 1985).

Whole body bioimpedance was performed by a phase sensitive single frequency device (BIA 101 BIVA, Akern srl, Florence, Italy), following standard procedures.

Vastus lateralis (VL) muscle architecture was evaluated at rest by a portable digital ultrasound system (Versana Active, GE HealthCare, Milwaukee, WI, USA), fitted with a 6–13 MHz, 4.7 cm linear array transducer. Measurements were performed on the VL muscle as previously described (Narici et al., 2021). Three different longitudinal images of the VL muscle were taken and saved for subsequent processing. The images obtained with this procedure were subsequently used to measure two different quantitative parameters, that is, muscle thickness (Tm) and fascicle length (Lf). The average of all Tm and Lf measures was considered for analysis, and the ratio between Lf and Tm was calculated to obtain an ultrasound index (USI) of the loss of muscle mass associated with sarcopenia (Narici et al., 2021).

#### 2.4.3 Microvascular/endothelial function

Microvascular/endothelial function was evaluated by determining the hyperaemic response in the common femoral artery during 1 min of passive leg movement (PLM) of a lower limb (Gifford & Richardson, 2017). Leg blood flow in the common femoral artery was evaluated by echo‐Doppler by multiplying blood flow velocity and vessel diameter, 2–3 cm proximal to the bifurcation (Versana Active), and a linear array transducer operating at an imaging frequency of 9 MHz. Measurements of the vessel diameter were taken from B‐mode images at the same point in the cardiac cycle (i.e., at the peak of the R wave of the simultaneously recorded integrated ECG). Blood velocities were recorded second‐by‐second by keeping the insonation angle at ≤60°, and the sample volume was centred and maximized according to the vessel lumen. Arterial blood flow was then calculated second‐by‐second. Participants remained seated with both legs fully extended and supported for a few minutes on a stool before resting blood flow data collection. The protocol consisted of 1 min of baseline data recording, followed by 1 min of passive knee extension and flexion. Participants’ lower leg was moved by a research team member through a 90° range of motion (180°–90°–180°) at a cadence of 1 Hz, following a metronome. The participants were instructed not to activate their muscles during the movements. Preliminary PLM trials were performed before the real measurements started. Measurements were performed in duplicate, with a 5‐min recovery period, and average values were obtained for each participant and retained for data analysis. Resting blood flow (mean value obtained during the baseline measurement), peak blood flow, the difference between peak and baseline blood flows (∆_peak_), and the area under the blood flow versus time response (area under the curve, AUC) were then calculated (Gifford & Richardson, 2017).

#### 2.4.4 Force measurements

Participants performed maximal voluntary contractions (MVC) of the right knee extensors while sitting upright on a custom‐built dynamometer, with both hips and knees flexed at 90° and with crossover chest straps to minimize movements of the trunk. Force was measured by a calibrated force cell (AM C3, Laumas Elettronica, Italy; sensitivity: 2.2 mV/V ±10%). Force data were converted into torque values by multiplying force by the lever arm. Torque data were recorded by AcqKnowledge software (BIOPAC Systems, Inc., Goleta, CA, USA).

#### 2.4.5 Constant work rate submaximal exercise

Participants completed a 6‐min constant work rate (CWR) exercise of moderate intensity on an electronically braked cycle ergometer (Monark 818E; Stockholm, Sweden). Intensity was set by selecting a work rate corresponding to ∼35% of the predicted peak oxygen uptake (${\dot{V}}_{O_{2}\mathrm{peak}}$). The corresponding work rate was then obtained by considering an O_2_ cost of exercise of 10 mL min^−1^ W^−1^ (Wasserman et al., 1999). During the test, respiratory parameters (i.e., pulmonary ventilation (${\dot{V}}_{E}$ ), tidal volume (*V*_T_ ), breathing frequency (*f*_R_), ${\dot{V}}_{O_{2}}$ and carbon dioxide output (${\dot{V}}_{CO_{2}}$), etc.) were assessed breath‐by‐breath by a metabolic cart (Quark PFTergo, Cosmed, Rome, Italy). Respiratory exchange ratio (RER) was calculated as ${\dot{V}}_{CO_{2}}$/${\dot{V}}_{O_{2}}$. Heart rate (HR) was recorded using 12‐lead electrocardiography (Quark C12x, Cosmed). Stroke volume (SV) and cardiac output (CO) were estimated by impedance cardiography (PhysioFlow; Manatec, Paris, France). By resolving the Fick equation, the arterial–mixed venous O_2_ concentration difference ($C_{\left(a-\bar{v}\right)O_{2}}$) was obtained from the direct measure of ${\dot{V}}_{O_{2}}$ and the estimate of CO. Arterialized blood O_2_ saturation ($S_{pO_{2}}$) was monitored by pulse oximetry (MicrO_2_; Siemens Medical Systems, Danvers, MA, USA) at the fingertip. At the end of the exercise, the rating of perceived exertion (RPE) was determined using the Borg 6–20 scale (Borg, 1973).

Oxygenation changes of VL of the right leg were determined by a portable continuous‐wave spatially resolved near infrared spectroscopy (NIRS) instrument (PortaMon; Artinis Medical System, Elst, The Netherlands). The instrument non‐invasively measures micromolar (µM) changes in oxygenated haemoglobin (Hb) + myoglobin (Mb) concentrations (∆[oxy(Hb+Mb)]) and in deoxygenated [Hb+Mb] (∆[deoxy(Hb+Mb)]), with respect to an initial value arbitrarily set equal to zero, obtained during the resting condition preceding the test. The sampling frequency was set at 10 Hz. An increased ∆[deoxy(Hb+Mb)] or a decreased ∆[oxy(Hb+Mb)] indicate an increased fractional O_2_ extraction in the tissue under consideration (Grassi & Quaresima, 2016). The ∆[deoxy(Hb+Mb)] signal was considered in the present study since it is usually less affected (with respect to the ∆[oxy(Hb+Mb)] signal) by changes in blood volume in the tissue (Grassi & Quaresima, 2016). The probe was firmly attached to the skin overlying the lower third of the quadriceps femoris muscle of the right thigh. The skin overlying the investigated muscle regions was carefully shaven and cleaned before the experiments. Adipose tissue thickness (ATT) at the site of NIRS probe application was measured by a calliper (Gima, Milan, Italy); a skinfold thickness of ≤20 mm was considered as the cut‐off value for NIRS utilization. Black bandages were put around the probe and the skin to prevent contamination from ambient light. ∆[deoxy(Hb+Mb)] data were expressed as a percentage of the maximal muscle deoxygenation obtained at rest by inflating a pressure cuff (∼300 mmHg) positioned at the inguinal crease of the thigh. This manoeuvre typically lasts a few minutes, until ∆[deoxy(Hb+Mb)] reaches a plateau, index of maximal fractional O_2_ extraction by skeletal muscle (Grassi & Quaresima, 2016).

Mean steady‐state values of the main variables determined during moderate‐intensity CWR exercise were determined during the last 20 s of exercise.

${\dot{V}}_{O_{2}}$ kinetics were evaluated during transitions from rest to moderate‐intensity CWR exercise. Breath‐by‐breath ${\dot{V}}_{O_{2}}$ values obtained during the 6‐min CWR exercise and the 4‐min CWR exercise preceding the incremental test (see below) were time aligned and then superimposed for each participant (Lamarra et al., 1987). ${\dot{V}}_{O_{2}}$ values were averaged every 10 s. Data obtained during the first 20 s of the transition (‘cardiodynamic’ phase) were excluded from analysis. Thus, ${\dot{V}}_{O_{2}}$ kinetics analysis dealt mainly with the ‘phase 2’ (or ‘fundamental’ component) of the response. To mathematically describe the ${\dot{V}}_{O_{2}}$ kinetics, data were fitted by the following function:

$$y\left(t\right)=y_{\mathrm{BAS}}+A_{f}\left[1-e^{-\left(t-{\mathrm{TD}}_{f}\right)/\tau_{f}}\right]$$

where *t* is time, $y_{\mathrm{BAS}}$ represents the baseline, $A_{f}\;$ is the amplitude between the *y*_BAS_ and the steady state during the fundamental component, $TD_{f}$ is the time delay, and $\tau_{f}$ the time constant of the function for the fundamental component. The O_2_ deficit was calculated by multiplying the mean response time (MRT = τ_f_ + TD_f_) and *A*_f_.

#### 2.4.6 Muscle O_2_ uptake recovery kinetics

In the recovery phase following the CWR cycling exercise, skeletal muscle ${\dot{V}}_{O_{2}}$ (${\dot{V}}_{O_{2}m}$) kinetics were evaluated in vivo by NIRS by the repeated occlusions method (Zuccarelli et al., 2020). ${\dot{V}}_{O_{2}m}$ was estimated during each occlusion by calculating the slope of the linear increase in ∆[deoxy(Hb+Mb)] during fifteen short (5 s) ischaemic periods induced by rapid inflation and deflation of a pneumatic cuff (DN 200/10/5, Stanley, New Britain, CT, USA). ${\dot{V}}_{O_{2}m}$ values were then plotted as a function of time and fitted by a monoexponential function as previously described by Ryan et al. (2014).

Resting ${\dot{V}}_{O_{2}m}$ was also estimated by calculating a linear regression of the ∆[deoxy(Hb+Mb)] increases during 5 s ischemia during the resting phase preceding the CWR exercise.

#### 2.4.7 Incremental exercise

Following an adequate recovery period (∼15 min), participants performed an incremental cycling exercise. Pedalling frequency was digitally displayed to the participants, who were asked to keep a constant cadence throughout the tests between 60 and 70 rpm. The protocol consisted of 5‐ to 10‐W increases every minute (depending on the participant's fitness level), preceded by 4 min at the same intensity of the CWR exercise, thereby allowing the participants to reach voluntary exhaustion (i.e., incapacity to maintain pedaling frequency despite verbal encouragement by the researchers) in 8–12 min. Cardio‐respiratory and metabolic responses to exercise were monitored as described above for the CWR. Gas exchange threshold (GET) was determined using both the ‘V‐slope’ method and ‘secondary criteria’ (Beaver et al., 1986); the respiratory compensation point (RCP) was identified by standard criteria (Wasserman & Whipp, 1975). Both GET and RCP were determined by two independent investigators. Mean values of ventilatory, pulmonary gas exchange, cardiovascular, and muscle oxygenation parameters were calculated during the last 20 s of each minute of exercise, and values obtained during the exhausting work rate were considered peak values. Ventilatory efficiency was calculated as the slope of ${\dot{V}}_{E}$ versus ${\dot{V}}_{CO_{2}}$ by excluding data above RCP. The higher the value, the more inefficient ventilation is in matching gas exchange with the pulmonary blood flow (Wasserman et al., 1999). The slope of ${\dot{V}}_{E}$ versus ${\dot{V}}_{CO_{2}}$ has been characterized in patients and in healthy subjects, and a value >34–35 is generally considered an index of impaired ventilatory efficiency (Chua et al., 1997; Kleber et al., 2000). The ratio between ${\dot{V}}_{E}/{\dot{V}}_{CO_{2}}$ at GET (${\dot{V}}_{E}/{\dot{V}}_{CO_{2}}$ at GET) was also evaluated, considering its lower variability compared to the slope calculation (Wasserman et al., 1999). Other cardiopulmonary variables such as ${\dot{V}}_{O_{2}}$ versus work rate and HR versus ${\dot{V}}_{O_{2}}$ were also determined.

### 2.5 Statistical analysis

Results are expressed as means ± SD values. A power analysis was performed a priori on the basis of the variance reported in a previous case–control study (Colosio et al., 2023) for the main variable of the present study, namely ${\dot{V}}_{O_{2}\mathrm{peak}}$ divided by unit of body mass. A sample size of 11 participants in each group was necessary to detect a statistically significant difference with an α value of 0.05 and a statistical power (1 − β) of 0.80 (G‐Power 3.1). Statistical significance of differences between the two groups (Long COVID versus Control) was checked by a two‐tailed Student's unpaired *t*‐test. Linear regression analysis and data fitting by exponential functions were carried out by the least‐squared residuals method. The level of significance was set at 0.05. Statistical analyses were carried out by a commercially available software package (Prism 8.0; GraphPad Software, San Diego, CA, USA).

## 3 RESULTS

Apart from the symptoms of fatigue, dyspnoea, muscle weakness and myalgia, which characterized the long COVID patients, the general characteristics of the two groups were not different (Table 1). The average interval between the onset of acute SARS‐CoV‐2 infection and the evaluation was 26 ± 5 months. Most of the patients were not hospitalized during the SARS‐CoV‐2 illness period of infection and reported a mild (presence of symptoms of acute upper respiratory tract infection) or moderate (pneumonia) illness.

### 3.1 Questionnaires

The IPAQ‐SF scores (physical activity level) were not different between groups (600 ± 344 and 635 ± 465 MET min week^−1^, in Control and Long COVID groups, respectively, *P* = 0.83). More specifically, 5/12 long COVID patients and 6/14 control patients gave the result ‘inactive’ (i.e., <600 MET min week^−1^), whereas all the others were ‘minimally active’ (i.e., >600 and < 3000 MET min week^−1^).

**FIGURE 1:**
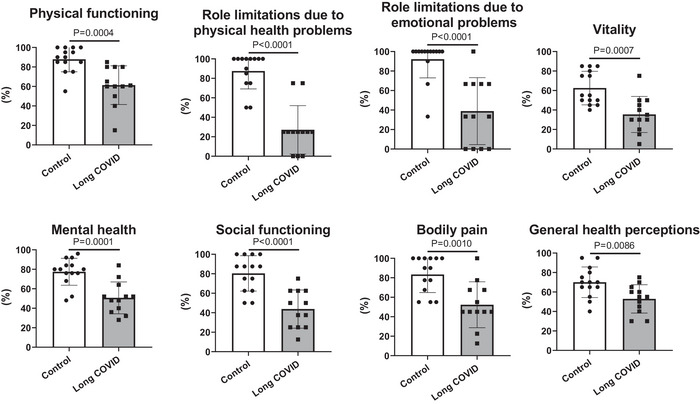
Mean (± SD) values of the eight general health domains (i.e., physical functioning, bodily pain, role limitations due to physical health problems, role limitations due to emotional problems, mental health, social functioning, vitality, and general health perceptions) covered by the MOS 36‐Item Short‐Form Health Survey (SF‐36) in patients with (Long COVID) or without (Control) persistent long COVID symptoms. Scores for each domain were transformed to a 0–100% scale, with a higher score defining a more favourable health state.

In Figure 1, the results of the SF‐36 questionnaire (evaluating the quality of life) are shown. In all investigated general health domains (i.e., physical functioning, bodily pain, role limitations due to physical health problems, role limitations due to emotional problems, mental health, social functioning, vitality and general health perceptions) scores were lower (*P* ≤ 0.01) in Long COVID versus Control patients, indicating a poorer quality of life in the first group.

### 3.2 Anthropometric measurements

Thigh volume and quadriceps femoris muscle mass, estimated by anthropometry, and body composition, evaluated by BIA, were not different between groups, as reported in Table 1. No differences were found between the two groups for the ultrasound sarcopenia index (USI) (Narici et al., 2021) (3.95 ± 0.65 in Control and 3.87 ± 0.64 in Long COVID; *P* = 0.75). According to the cut‐off values for the stratification of sarcopenia (Narici et al., 2021), a prevalence of non‐sarcopenic profiles (∼60% of patients) was observed in both groups; ∼35–40% of patients were pre‐sarcopenic, and only one patient in the Control group was moderately sarcopenic.

### 3.3 Force measurements

Isometric MVC of knee extensors, expressed as torque, was similar between groups (141 ± 59 N m in Control and 141 ± 62 N m in Long COVID; *P* = 0.99).

### 3.4 Microvascular/endothelial function

**FIGURE 2:**
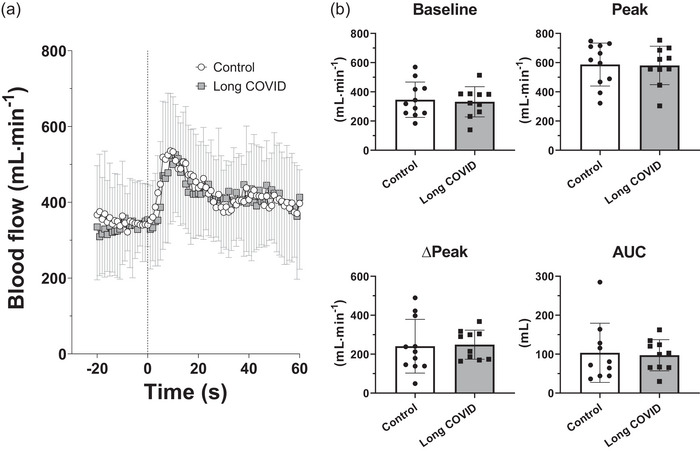
(a) Mean values (±SD) of blood flow in the common femoral artery at rest and in response to 1‐min passive leg movement (PLM), in patients with some persistent long COVID symptoms (Long COVID) and in control participants (Control). The vertical dashed line indicates the onset of PLM. (b) Individual and mean (±SD) values of baseline blood flow (Baseline), peak blood flow (Peak), changes from baseline to peak blood flow (∆_peak_) and area under the blood flow versus time curve (AUC) in response to 1‐min PLM, in patients with some persistent long COVID symptoms (Long COVID, *n* = 10) and in control participants (Control, *n* = 11).

Common femoral artery diameter was not different in Control (0.91 ± 0.12 cm) and Long COVID (0.93 ± 0.12 cm) patients (*P* = 0.73). Mean (±SD) values of leg blood flow obtained at rest and during 1 min of PLM and individual and mean (±SD) values of the main parameters evaluated during PLM are shown in Figure 2. Baseline blood flow was not different in the two groups (*P* = 0.78). Leg blood flow increased immediately after the onset of the PLM, reaching a similar peak (*P* = 0.92) after about 10 s in both groups. No differences were found in Control versus Long COVID patients for the difference between peak and baseline blood flows (∆_peak_) (*P* = 0.87), or for the area under the blood flow versus time curve (AUC) (*P* = 0.82).

### 3.5 Constant work rate submaximal exercise

Mean (±SD) steady‐state values of the main variables obtained at the end of the CWR exercise are given in Table 2. Work rate corresponded to 72 ± 9% and 73 ± 11% of GET in Control and Long COVID patients, respectively (*P* = 0.78), and was within the moderate intensity domain (i.e., <GET) for all participants. No differences were observed between groups, except for *f*_R_ (*P* = 0.006) and RPE (*P* = 0.02), which were higher in Long COVID (*f*_R_ 23.6 ± 3.9 b min^−1^; RPE 11 ± 2) than in Control patients (*f*_R_ 19.4 ± 3.4 b min^−1^; RPE 9 ± 2).

**TABLE 2:** Steady‐state mean values (± SD) of the main variables obtained at the end of the moderate‐intensity CWR exercise, in control participants (Control) and in patients with some persistent long COVID symptoms (Long COVID).

	Control	Long COVID	*P*
Work rate (W)	38 ± 13	38 ± 17	0.98
${\dot{V}}_{O_{2}}$ (L min^−1^)	0.903 ± 0.241	0.907 ± 0.244	0.97
${\dot{V}}_{O_{2}}$ (mL kg min^−1^)	12.2 ± 2.3	11.1 ± 1.7	0.21
${\dot{V}}_{CO_{2}}$ (L min^−1^)	0.773 ± 0.205	0.767 ± 0.204	0.94
RER	0.86 ± 0.06	0.85 ± 0.05	0.66
${\dot{V}}_{E}$ (L min^−1^)	25.8 ± 6.0	28.4 ± 7.9	0.34
*V*_T_ (L)	1.364 ± 0.337	1.248 ± 0.381	0.42
*f*_R_ (breaths min^−1^)	19.4 ± 3.4	23.6 ± 3.9**	0.006
$P_{\mathrm{ET}O_{2}}$ (mmHg)	101.9 ± 4.0	104.9 ± 4.8	0.10
$P_{\mathrm{ET}O_{2}}$ (mmHg)	39.4 ± 2.8	36.8 ± 4.4	0.09
HR (b min^−1^)	102 ± 17	96 ± 12	0.26
HR (% HR_peak_)	65 ± 8	63 ± 8	0.64
SV (mL)	82.5 ± 25.3	83.5 ± 14.9	0.92
CO (L min^−1^)	8.2 ± 2.3	7.7 ± 1.6	0.63
$C_{\left(a-\bar{v}\right)O_{2}}$ (mL dL^−1^)	12.4 ± 3.1	11.7 ± 3.3	0.64
$S_{pO_{2}}$ (%)	98 ± 2	97 ± 1	0.27
∆[deoxy(Hb+Mb)] (% ischaemia)	12.8 ± 9.0	16.0 ± 7.2	0.53
RPE (6–20)	9 ± 2	11 ± 2*	0.02

*Note*: Data are means ± SD. Asterisks denote differences between groups by means of Student's unpaired *t*‐test: *P < 0.05; **P < 0.01. Abbreviations: $C_{\left(a-\bar{v}\right)O_{2}}$, arteriovenous O_2_ difference; *f*_R_, breathing frequency; HR, heart rate; $P_{\mathrm{ETC}O_{2}}$, end‐tidal CO_2_ partial pressure; $P_{\mathrm{ET}O_{2}}$, end‐tidal O_2_ partial pressure; RER, respiratory exchange ratio; RPE, rating of perceived exertion obtained during the moderate‐intensity constant work rate (CWR) exercise; $S_{pO_{2}}$, arterialized blood O_2_ saturation by pulse oximetry; SV, stroke volume; ${\dot{V}}_{E}$, pulmonary ventilation; ${\dot{V}}_{CO_{2}}$, CO_2_ output; ${\dot{V}}_{O_{2}}$, pulmonary O_2_ uptake; *V*_T_, tidal volume; ∆[deoxy(Hb+Mb)], deoxygenated Hb+Mb concentrations in vastus lateralis muscle.

In terms of the ${\dot{V}}_{O_{2}}$ kinetics during the transition from rest to the moderate‐intensity CWR exercises, $y_{\mathrm{BAS}}\;$ was 0.257 ± 0.071 (95% confidence interval (CI) 0.216–0.297) and 0.238 ± 0.058 L min^−1^ (95% CI 0.201–0.275) in Control and Long COVID, respectively, whereas $A_{f}$ was 0.659 ± 0.191 L min^−1^ (95% CI 0.548–0.769) in Control and 0.650 ± 0.198 L min^−1^ (95% CI 0.524–0.775) in Long COVID. No differences were observed between groups for the above‐mentioned variables, as well as for $\tau_{f}$, which was 36.3 ± 9.1 s (95% CI 31.0–41.5) and 34.3 ± 8.1 s (95% CI 29.2–39.4) in Control and Long COVID, respectively (*P* = 0.56). Accordingly, the O_2_ deficit was not different between groups (0.469 ± 0.186 L (95% CI 0.362–0.577) in Control and 0.410 ± 0.100 L (95% CI 0.343–0.478) in Long COVID; *P* = 0.35).

### 3.6 Muscle O_2_ uptake recovery kinetics

**FIGURE 3:**
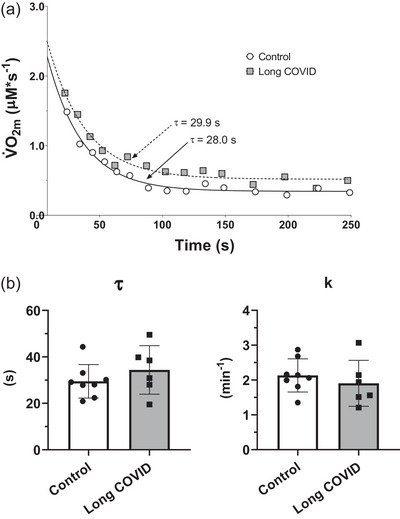
(a) NIRS‐obtained muscle ${\dot{V}}_{O_{2}}$ (${\dot{V}}_{O_{2}m}$) recovery kinetics for a representative participant for each group (Control and Long COVID) following the moderate‐intensity constant work rate exercise. The fitted functions and the calculated time constant (τ) values are also shown. (b) Individual and mean (± SD) values of τ (time constant) and *k* (velocity constant, *k* = 1/τ), in patients with persistent long COVID symptoms (Long COVID, *n* = 6) and in control participants (Control, *n* = 8).

Representative ${\dot{V}}_{O_{2}m}$ recovery kinetics curves evaluated in vivo by near‐infrared spectroscopy (NIRS) by the repeated occlusions method in a typical Control and in a typical Long COVID patient are shown in Figure 3a. For all experiments, the fitting was excellent, and the coefficients of determination (*r* ^2^) were >0.90.

Skinfold thickness of the participants (*n* = 14) in which the NIRS measurements were performed was ∼8.9 ± 4.6 mm; the remaining participants, almost exclusively females, had a relatively thick skinfold (25.8 ± 4.0 mm), which precluded NIRS measurements (Grassi & Quaresima, 2016). Individual and mean (± SD) values of τ (time constant) and *k* (velocity constant, *k* = 1/τ) of the ${\dot{V}}_{O_{2}m}$ recovery kinetics are shown in Figure 3b. Neither τ (*P* = 0.32) nor *k* (*P* = 0.47) was different in Long COVID versus Control patients.

In both groups ${\dot{V}}_{O_{2}m}$ values at the onset of recovery following CWR exercise (values extrapolated at time = 0 according to the fitted monoexponential curves) were about 17 times higher than those determined at rest. Also, resting ${\dot{V}}_{O_{2}m}$ values were not different between groups (*P* = 0.90).

### 3.7 Incremental exercise

Values of the main respiratory, cardiovascular and metabolic variables obtained during the incremental exercise are shown in Table 3.

**TABLE 3:** Mean ± SD values of the main respiratory, cardiovascular and metabolic variables determined during the incremental exercise in patients with some persistent long COVID symptoms (Long COVID) and in patients without these symptoms (Control).

	Control	Long COVID	*P*
Peak work rate (W)	146 ± 52	125 ± 53	0.32
Peak work rate (W/kg)	1.95 ± 0.48	1.58 ± 0.54	0.08
${\dot{V}}_{O_{2}\mathrm{peak}}$ (L min^−1^)	2.030 ± 0.710	1.807 ± 0.515	0.38
${\dot{V}}_{O_{2}\mathrm{peak}}$ (mL kg^−1^ min^−1^)	26.6 ± 6.3	23.0 ± 4.9	0.12
${\dot{V}}_{O_{2}\mathrm{peak}}$ (% predicted)	105 ± 19	97 ± 19	0.32
${\dot{V}}_{O_{2}}$/work rate slope (mL min^−1^ W^−1^)	10.7 ± 0.9	11.0 ± 1.1	0.45
${\dot{V}}_{CO_{2}\mathrm{peak}}$ (L min^−1^)	2.265 ± 0.853	1.902 ± 0.533	0.21
RER_peak_	1.11 ± 0.09	1.06 ± 0.05	0.12
${\dot{V}}_{\mathrm{Epeak}}$ (L min^−1^)	70.8 ± 28.9	69.0 ± 20.4	0.86
*V*_Tpeak_ (L)	2.3 ± 0.7	1.9 ± 0.6	0.21
*f*_Rpeak_ (breaths min^−1^)	31.8 ± 5.0	37.2 ± 8.0	0.054
${P_{\mathrm{ET}O_{2}}}_{\mathrm{peak}}$ (mmHg)	111.7 ± 3.5	112.8 ± 5.2	0.55
${P_{\mathrm{ET}O_{2}}}_{\mathrm{peak}}$ (mmHg)	37.9 ± 3.2	35.0 ± 5.3	0.08
EqO_2peak_	35.0 ± 3.4	37.0 ± 6.0	0.30
EqCO_2peak_	31.6 ± 2.4	35.7 ± 5.6*	0.021
${\dot{V}}_{E}/{\dot{V}}_{CO_{2}}$ slope	27.5 ± 3.2	32.8 ± 6.0**	0.008
${\dot{V}}_{E}/{\dot{V}}_{CO_{2}}$ at GET	30.7 ± 2.7	34.5 ± 4.7*	0.017
${\dot{V}}_{O_{2}\mathrm{GET}}$ (L min^−1^)	1.277 ± 0.420	1.282 ± 0.373	0.97
${\dot{V}}_{O_{2}\mathrm{GET}}$ (%${\dot{V}}_{O_{2}\mathrm{peak}}$)	65 ± 9	69 ± 6	0.09
${\dot{V}}_{O_{2}\mathrm{RCP}}$ (L min^−1^)	1.732 ± 0.673	1.645 ± 0.453	0.67
${\dot{V}}_{O_{2}\mathrm{RCP}}$ (%${\dot{V}}_{O_{2}\mathrm{peak}}$)	85 ± 7	89 ± 6	0.17
HR_peak_ (b min^−1^)	159.0 ± 15.4	152.0 ± 12.3	0.21
HR_peak_ (% predicted)	93 ± 8	90 ± 7	0.33
HR/${\dot{V}}_{O_{2}}$ slope (b L^−1^)	49.5 ± 10.9	56.8 ± 13.0	0.13
SV_peak_ (mL)	108.5 ± 25.2	105.1 ± 22.1	0.79
CO_peak_ (L min^−1^)	15.8 ± 5.1	15.6 ± 2.9	0.96
CO_peak_/${\dot{V}}_{O_{2}\mathrm{peak}}$	7.7 ± 3.1	8.8 ± 1.2	0.43
${C_{\left(a-\bar{v}\right)O_{2}}}_{\mathrm{peak}}$ (mL dL^−1^)	11.6 ± 1.6	11.4 ± 2.7	0.89
${S_{pO_{2}}}_{\mathrm{peak}}$ (%)	98 ± 2	98 ± 1	0.61
∆[deoxy(Hb+Mb)]_peak_ (% ischaemia)	44.3 ± 17.2	40.7 ± 12.6	0.67
RPE_peak_ (6–20)	17 ± 2	17 ± 1	0.79

*Note*: Data are means ± SD. *P*‐values relate to differences between groups by means of Student's unpaired *t*‐test: *P < 0.05; **P < 0.01. Abbreviations: Work rate; $C_{\left(a-\bar{v}\right)O_{2}}$, arteriovenous O_2_ difference; CO, cardiac output; EqCO_2_, ventilatory equivalents for CO_2_; EqO_2_, ventilatory equivalents for O_2_; *f*_R_, breathing frequency; HR, heart rate; $P_{\mathrm{ETC}O_{2}}$, end‐tidal CO_2_ partial pressure; $P_{\mathrm{ET}O_{2}}$, end‐tidal O_2_ partial pressure; RER, respiratory exchange ratio; $S_{pO_{2}}$, arterialized blood O_2_ saturation by pulse oximetry; SV, stroke volume; ${\dot{V}}_{CO_{2}}$, carbon dioxide (CO_2_) output; ${\dot{V}}_{E}$, pulmonary ventilation; ${\dot{V}}_{O_{2}}$, pulmonary oxygen uptake; *V*_T_, tidal volume; ${\dot{V}}_{O_{2}\mathrm{GET}}$, pulmonary O_2_ uptake at GET; ${\dot{V}}_{O_{2}\mathrm{RCP}}$, pulmonary O_2_ uptake at RCP; ∆[deoxy(Hb+Mb)], skeletal muscle fractional O_2_ extraction; RPE, rating of perceived exertion.

No significant differences were found in Long COVID versus Control patients for the variables evaluating maximal cardiorespiratory function, such as peak work rate, ${\dot{V}}_{O_{2}\mathrm{peak}}$, HR_peak_, CO_peak_, ${S_{pO_{2}}}_{\mathrm{peak}}$ and maximal peripheral O_2_ extraction (${C_{\left(a-\bar{v}\right)O_{2}}}_{\mathrm{peak}}$ and ∆[deoxy(Hb+Mb)]_peak_). RER_peak_ and HR_peak_ values suggest that the tests were close to maximal in both groups. Also, for the ‘ventilatory threshold’ variables, evaluating the fraction of ${\dot{V}}_{O_{2}\mathrm{peak}}$ which can be sustained for relatively long periods of time (GET and RCP), no significant differences were observed between the two groups.

Individual ${\dot{V}}_{O_{2}}$ values were plotted as a function of the corresponding work rate during the incremental test, and regression lines were drawn. The ${\dot{V}}_{O_{2}}$ versus work rate slope was 11.0 ± 1.1 and 10.7 ± 0.9 mL min^−1^ W^−1^ in Long COVID and in Control patients, respectively, with no differences between groups (*P* = 0.45). HR versus ${\dot{V}}_{O_{2}}$ slopes were also not different between groups (*P* = 0.13).

**FIGURE 4:**
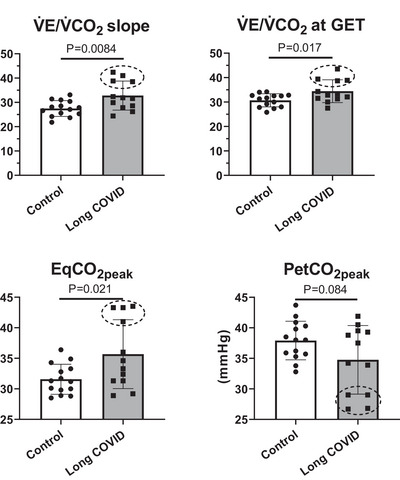
Individual and mean (±SD) values of the slope of pulmonary ventilation versus CO_2_ output (${\dot{V}}_{E}/{\dot{V}}_{CO_{2}}$ slope) below RCP, ${\dot{V}}_{E}/{\dot{V}}_{CO_{2}}$ at the gas exchange threshold (${\dot{V}}_{E}/{\dot{V}}_{CO_{2}}$ at GET), ventilatory equivalents for CO_2_ (EqCO_2_) and end‐tidal CO_2_ partial pressure ($P_{\mathrm{ETC}O_{2}}$) at peak exercise in patients with some persistent long COVID symptoms (Long COVID) and in patients with no symptoms (Control). A subgroup (*n* = 4) of Long COVID patients with higher values of ${\dot{V}}_{E}/{\dot{V}}_{CO_{2}}$ slope and EqCO_2_ and a trend towards lower values of $P_{\mathrm{ETC}O_{2}}$ compared to the rest of patients is circled with a dotted line.

In terms of ventilatory variables, some differences between groups were observed. Whereas ${\dot{V}}_{\mathrm{Epeak}}$ values were almost identical in the two groups, *f*_Rpeak_ (at the limit of statistical difference), ventilatory equivalent for CO_2_ [EqCO_2peak_], ${\dot{V}}_{E}/{\dot{V}}_{CO_{2}}$ at GET and the ${\dot{V}}_{E}/{\dot{V}}_{CO_{2}}$ slope calculated below RCP were significantly higher in Long COVID versus Control patients. Individual patients’ values (Figure 4) show that the difference between Long COVID and Control patients was substantially attributable to a subgroup of four Long COVID patients (two males and two females), who showed significantly elevated values of EqCO_2peak_ (42.8 ± 1.2), ${\dot{V}}_{E}/{\dot{V}}_{CO_{2}}$ slope (40.2 ± 1.8), ${\dot{V}}_{E}/{\dot{V}}_{CO_{2}}$ at GET (40.2 ± 2.3) and significantly low values of ${P_{\mathrm{ETC}O_{2}}}_{\mathrm{peak}}$ (27.9 ± 1.3 mmHg).

**FIGURE 5:**
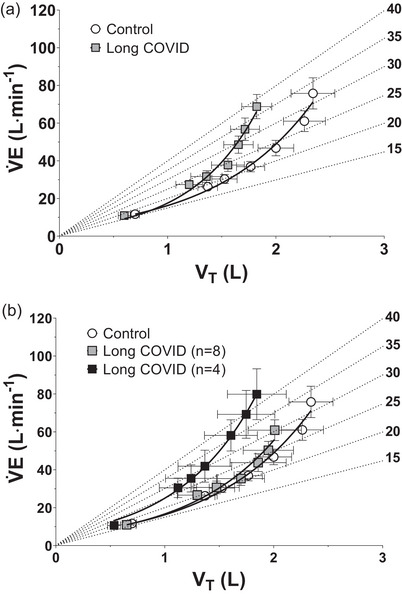
(a) Mean (± SEM) values of pulmonary ventilation (${\dot{V}}_{E}$) (grouped for discrete tidal volume (*V*_T_) intervals) as a function of *V*_T_ in Control and Long COVID during the incremental exercise. (b) The ventilatory pattern for Long COVID patients with ventilatory inefficiency (i.e., high ${\dot{V}}_{E}/{\dot{V}}_{CO_{2}}$ slope, *n* = 4) was also shown. Iso‐respiratory frequency (*f*_R_) lines (dashed lines, departing from the origin) and the exponential functions fitting the data points were also reported in both graphs.

In order to check possible differences in the ventilatory pattern (the relative contribution of *V*_T_ and *f*_R_ increases to the ${\dot{V}}_{E}$ increase), the latter was specifically investigated by the analysis depicted in Figure 5, in which mean values obtained during the incremental exercise are presented. In Figure ${\dot{V}}_{E}$ data were plotted as a function of *V*_T_, and iso‐*f*_R_ lines were drawn. Figure 5a shows a different ventilatory pattern between groups, with a steeper ${\dot{V}}_{E}$/*V*_T_ relationship in Long COVID versus Control patients (greater contribution of *f*_R_ increase, compared to the *V*_T_ increase, for the same ${\dot{V}}_{E}$). Figure 5b shows the ventilatory pattern of the subgroup of Long COVID patients with ventilatory inefficiency (Chua et al., 1997; Kleber et al., 2000) (i.e., high ${\dot{V}}_{E}/{\dot{V}}_{CO_{2}}$ slope, *n* = 4, see above) is shown, and a steeper ${\dot{V}}_{E}$/*V*_T_ compared to the other Long COVID patients and the Control patients was evident. Therefore, also for the ventilatory pattern, the observed group differences (Long COVID versus Control patients) were substantially attributable to the four long COVID patients with ventilatory inefficiency.

Besides ventilatory inefficiency and an altered ventilatory pattern, these four long COVID patients did not exhibit other distinctive characteristics related to the other variables (e.g., symptoms, level of physical activity, muscle mass or force, variables evaluating exercise tolerance or microvascular/endothelial function).

## 4 DISCUSSION

In the present study, carried out on patients more than 2 years after a relatively mild COVID‐19 illness, a clear discrepancy was observed between the persistence, in some patients, of symptoms of fatigue/exercise intolerance, such as excessive or early fatigue, excessive or early dyspnoea on exertion, muscle weakness and myalgias (belonging to the long COVID syndrome; Davis et al., 2023; Soriano et al., 2022), and the absence of several pathophysiological mechanisms potentially responsible for the symptoms. The habitual level of physical activity, muscle size and strength, maximal aerobic power and the ventilatory thresholds, the kinetics of pulmonary ${\dot{V}}_{O_{2}}$, peak cardiac function, the mechanical efficiency of cycling, microvascular/endothelial function, skeletal muscle oxidative metabolism and mitochondrial respiration evaluated in vivo (muscle ${\dot{V}}_{O_{2}}$ recovery kinetics) were indeed not different between post‐COVID patients with (‘Long COVID’ group) and without (‘Control’ group) the symptoms mentioned above. The only exception was represented by evidence of ventilatory inefficiency in a subgroup of Control patients. The discrepancy between the presence of symptoms and the absence of pathophysiological mechanisms, among those investigated in the present study, was not observed in previous studies with shorter follow‐up periods after the original infection (Appelman et al., 2024; Colosio et al., 2023; Ramírez‐Vélez et al., 2023; Singh et al., 2022; Szekely et al., 2021).

Thus, more than 2 years after the original infection, the potential pathophysiological mechanisms responsible for fatigue and other exercise‐intolerance‐related symptoms in long COVID patients are unclear.

The mechanisms may reside in the neurological and/or psychological domain, which were not specifically investigated in the present study. The persistence of fatigue was already reported by Colizzi et al. (2024), who observed a prevalence of ∼40% of the symptoms at a 2‐year follow‐up after an acute COVID‐19 infection in a cohort of 230 patients, which included the patients studied in the present study. Moreover, in the same study (Colizzi et al., 2024) several neuropsychiatric symptoms were reported in the patients. Long COVID is indeed well known to determine impairments which include anxiety/depression, cognitive impairment, neuropathies, chronic fatigue syndrome, dysfunctional neurological signalling, dysautonomia, etc. (Davis et al., 2023; Kedor et al., 2022). It has been recently hypothesized (Thomas et al., 2024) that in long COVID patients, impaired sensory processing might increase effort perception, leading to fatigue (and possibly other symptoms), which could be the result of complex interactions between physiological and psychological factors. This could explain, at least in part, the discrepancy between the reported symptoms (which translated into poorer scores on a questionnaire evaluating the quality of life) and the substantial absence of pathophysiological mechanisms. From the results of the present study, however, we cannot exclude that other pathophysiological mechanisms not specifically investigated in the study, such as chronic systemic inflammation, autonomic dysregulation, immune dysregulation, viral persistence and coagulopathies, may play a role in the pathophysiology of symptoms in long COVID patients (Sherif et al., 2023; Soares et al., 2022).

In the present study we conducted an extensive series of measurements aimed at investigating if some pathophysiological mechanisms, proposed in recent years in order to explain the signs and symptoms of the long COVID syndrome in patients evaluated during the first few months following the original infection, could be identified also in the patients after a significantly longer (i.e., more than 2 years) follow‐up. As mentioned above, the answer to this question was mostly negative. Even when the investigated variables (see below) identified impairments with respect to reference values obtained in healthy subjects, no significant differences were observed between Long COVID and Control patients.

The symptoms could not be ascribed to deconditioning, as suggested for shorter follow‐ups by Rinaldo et al. (2021). The habitual level of physical activity in our patients was in the ‘minimally active’ or ‘inactive’ range, with no difference between the two groups.

The symptoms could not be attributable to reduced muscle mass or force, as suggested for shorter follow‐up by Ramirez‐Veléz et al. (2023). In our study, lower limb muscle volume (no signs of sarcopenia were identified, with the exception of one patient of the Control group) and muscle strength values were not different in the two groups.

Variables evaluating exercise tolerance, such as ${\dot{V}}_{O_{2}\mathrm{peak}}$ and the ‘ventilatory thresholds’ (i.e., the ‘gas exchange threshold’ (GET) and the ‘respiratory compensation point’ (RCP)) were not different in the two groups. ${\dot{V}}_{O_{2}\mathrm{peak}}$ is an index of the maximal power which can be sustained by aerobic metabolism, and it evaluates the maximal performance of the integrated responses of the respiratory, cardiovascular, and skeletal muscle systems to exercise. Perhaps even more importantly, ${\dot{V}}_{O_{2}\mathrm{peak}}$ is an index of ‘cardiorespiratory fitness’, strongly associated with all‐cause mortality (Blair et al., 1989). GET and RCP, on the other hand, indicate fractions of ${\dot{V}}_{O_{2}\mathrm{peak}}$ which can be sustained for relatively longer periods of time compared to ${\dot{V}}_{O_{2}\mathrm{peak}}$. In most previous studies ${\dot{V}}_{O_{2}\mathrm{peak}}$, and (when these variables were determined) ventilatory thresholds were significantly lower in patients with long COVID symptoms versus patients without the symptoms 2–14 months (Aparisi et al., 2021; Colosio et al., 2023; Jamieson et al., 2024; Longobardi et al., 2022; Pleguezuelos et al., 2021; Singh et al., 2022; Szekely et al., 2021) and ∼1.5 years (Appelman et al., 2024) after the initial SARS‐CoV‐2 infection. These observations were not confirmed in the present study after a >2 year follow‐up. Our results support those by Berg et al. (2024), Noureddine et al. (2023), and Dorelli et al. (2024) who reported normal ${\dot{V}}_{O_{2}\mathrm{peak}}$ values (i.e., ${\dot{V}}_{O_{2}\mathrm{peak}}$ > 85% of predicted) and/or values not different from healthy controls in long COVID patients 9, 12 and 34 months after SARS‑CoV‑2 infection, respectively.

Variables evaluating peak cardiac function (HR_peak_, SV_peak_, CO_peak_) were not different in the two groups. The same result was reported in previous studies (Appelman et al., 2024; Colosio et al., 2023; Singh et al., 2022), 8 months to ∼1.5 years after recovery from an acute illness. Cardiac performance, on the other hand, was impaired in patients with long COVID symptoms evaluated after shorter follow‐ups (i.e., 3 months after onset of COVID‐19 symptoms) by Szekely et al. (2021).

The variables evaluating microvascular/endothelial function, determined during the PLM test, were not different in the two groups of patients. The value of the AUC of the blood flow versus time profile, and from the difference between baseline and peak blood flow (∆_peak_) were lower than those obtained, by the same method, in a group of slightly older (73 ± 2 years) healthy sedentary individuals reported by Groot et al. (2016). In other words, the microvascular/endothelial function was somehow impaired in our patients, but with no difference between Long COVID and Control patients. Impairments of microvascular function were described by previous authors (sometimes by utilizing different methods) in long COVID patients evaluated during shorter follow‐ups (Jamieson et al., 2024; Nandadeva et al., 2021; Paneroni et al., 2021; Ratchford et al., 2021). Microvascular and endothelial dysfunction is critical in the pathogenesis of COVID disease, as it contributes to the pro‐coagulant, pro‐inflammatory and pro‐oxidant state impairing peripheral O_2_ delivery and utilization (Davis et al., 2023; Serviente et al., 2022). In sum, our PLM data suggest that a microvascular/endothelial dysfunction was still present in the patients more than 2 years after the original infection, but it was likely not directly responsible for the symptoms in the patients of the Long COVID group.

Mechanical efficiency (see the slope of ${\dot{V}}_{O_{2}}$ versus work rate during the incremental exercise), as well as the pulmonary ${\dot{V}}_{O_{2}}$ on‐kinetics and the size of the O_2_ deficit, was not different in the two groups of patients. This is in contrast with previous studies, reporting a lower mechanical efficiency (Pleguezuelos et al., 2021; Rinaldo et al., 2021) and a slower pulmonary ${\dot{V}}_{O_{2}}$ kinetics (Colosio et al., 2023) together with a greater O_2_ deficit (Longobardi et al., 2022) in long COVID patients observed after shorter follow‐ups. On the other hand, Berg et al. (2024) reported a similar walking economy between patients with long COVID and healthy controls 9 months after the onset of acute COVID‐19 illness.

In terms of skeletal muscle oxidative metabolism, in the present study, two main variables of functional evaluation were investigated. Peak fractional O_2_ extraction data, evaluated during the incremental exercise by NIRS, were not different in the two groups of patients (see Table 3). On the contrary, a reduced peripheral O_2_ extraction was observed during exercise by Colosio et al. (2023) and Appelman et al. (2024) in patients with long COVID symptoms versus healthy controls during relatively shorter follow‐ups. Also, the kinetics of muscle O_2_ uptake (${\dot{V}}_{O_{2}m}$) recovery following exercise, which we estimated by NIRS and the repeated ischaemia approach, was not different between the two groups of patients (see Figure 3). This variable represents a ‘mirror image’ of muscle phosphocreatine recovery kinetics, and is considered a reference method for evaluating skeletal muscle oxidative metabolism (Zuccarelli et al., 2020). Also, this variable was found to be impaired in long COVID patients following shorter follow‐ups (Colosio et al., 2023).

For only one aspect, a significant difference was observed between Long COVID and Control patients, that is, for variables evaluating ventilatory inefficiency (Sun et al., 2002). More specifically, the ${\dot{V}}_{E}/{\dot{V}}_{CO_{2}}$ slope (determined during the incremental exercise before RCP), the ratio between ${\dot{V}}_{E}$ and ${\dot{V}}_{CO_{2}}$ evaluated at GET (${\dot{V}}_{E}/{\dot{V}}_{CO_{2}}$ at GET), and the ventilatory equivalent for CO_2_ (EqCO_2_) determined at peak exercise were higher in Long COVID versus Control patients. End‐tidal O_2_ partial pressure ($P_{\mathrm{ET}O_{2}}$) at peak exercise showed a trend towards lower values in the long COVID patients. These data confirm previous observations in long COVID patients evaluated after shorter follow‐ups (Baratto et al., 2021; Dorelli et al., 2021). The long COVID patients in the present study showed a different ventilatory pattern versus the Control patients. Whereas ${\dot{V}}_{\mathrm{Epeak}}$ values were almost identical in the two groups, long COVID patients had significantly higher *f*_Rpeak_ and a trend towards significantly lower *V*_Tpeak_ (see Table 3). A different ventilatory pattern, with a greater contribution of *f*_R_ and a smaller contribution of *V*_T_ for the same ${\dot{V}}_{E}$, was evident in the long COVID patients also at submaximal work rates (see Figure 5). An increased *f*_R_ was observed in the long COVID group also at the end of the moderate‐intensity CWR exercise, in association with a higher RPE (see Table 2), which could be an indication of a greater ‘subjective fatigability’, whose causes may lie in domains not specifically investigated in the present study.

Individual values of ${\dot{V}}_{E}/{\dot{V}}_{CO_{2}}$ slopes and ${\dot{V}}_{E}/{\dot{V}}_{CO_{2}}$ at GET are shown in Figure 4. As a reference, for subjects of 51–60 years of age, the upper limit of the normal values for these variables should be around 31 (Sun et al., 2002). In chronic heart failure patients higher than normal ${\dot{V}}_{E}/{\dot{V}}_{CO_{2}}$ slopes are associated with a worse prognosis and higher mortality rates (Kleber et al., 2000). In the present study, the higher values in long COVID patients were attributable only to four patients (two males and two females), indicated in Figure 4 by the circles. In this subgroup, the mean (±SD) values of ${\dot{V}}_{E}/{\dot{V}}_{CO_{2}}$ slope (40.2 ± 1.8) and ${\dot{V}}_{E}/{\dot{V}}_{CO_{2}}$ at GET (40.2 ± 2.3) were well above the normal range. A greater than normal ${\dot{V}}_{E}/{\dot{V}}_{CO_{2}}$ slope (or ${\dot{V}}_{E}/{\dot{V}}_{CO_{2}}$ at GET) indicates an inefficiency of pulmonary ventilation and gas exchange, possibly related to an intrapulmonary mismatch of alveolar ventilation to lung perfusion (Dorelli et al., 2024; Singh et al., 2022; Sun et al., 2002). Alveolar ventilation–lung perfusion mismatch is a key pathophysiological mechanism responsible for the hypoxaemia which can occur in the acute phase of the COVID‐19 infection (Habashi et al., 2021). According to some studies, breathing abnormalities in long COVID patients could be related to an aberrant ventilatory control, by either a stimulation of activator systems (automatic and cortical ventilatory control, peripheral afferents, and sensory cortex) or a suppression of inhibitory systems (endorphins) (Motiejunaite et al., 2020). Also, another potential mechanism could be an autonomic dysfunction, as ventilatory inefficiency have also been linked to and altered parasympathetic nervous system function in patients with long COVID (Aparisi et al., 2021; Dorelli et al., 2021). Besides these aspects, our data suggest that the ventilatory inefficiency may persist, in a minority of symptomatic patients, years after the original infection, despite normal ${\dot{V}}_{O_{2}\mathrm{peak}}$ values, confirming recent observations by Dorelli et al. (2024) and by Noureddine et al. (2023). Interestingly, the subgroup of patients with ventilatory inefficiency were not different than the other patients of the study in terms of symptoms or other functional variables. This topic definitively needs further studies.

The described ventilatory inefficiency could be the target of specific interventions, such as respiratory muscle training (Nazir & Hasri, 2022).

The present study has some limitations. The number of patients was relatively limited. The study was specifically powered a priori to determine differences for the main variable of the study (${\dot{V}}_{O_{2}\mathrm{peak}}$) (see ‘Statistics’), but not in secondary outcomes. A greater number of patients would also have allowed a greater emphasis on subgroup analyses (see e.g., above, the issue of ventilatory inefficiency).

The inferences we made on symptoms and pathophysiological mechanisms apply only to the type of patient selected for the study, that is, patients tested more than 2 years after the first outbreak of the pandemic disease, originally affected by a relatively mild disease, and who underwent ≥2 vaccinations. In this respect, the present study represents a ‘snapshot’ of a subgroup of the very heterogeneous population affected by COVID‐19.

The lack of longitudinal data obtained in the same patients before acute infection or after shorter follow‐up periods represents another limitation, since it did not allow us to confirm if some impairments (as suggested by the literature) might actually have been present after more than 2 years, or at an earlier phase following the original infection. The lack of longitudinal data also did not allow us to identify a ‘reversal’ of some pathophysiological mechanisms during the follow‐up.

In the present study, we did not determine the presence of post‐exertional malaise, one of the cardinal and distinctive symptoms of myalgic encephalomyelitis/chronic fatigue syndrome, and a common symptom also among patients with long COVID (Appelman et al., 2024). It should be noted, however, that post‐exertional malaise was not considered in several other recent papers dealing with long Covid syndrome (Colosio et al., 2023; Noureddine et al., 2023; Ramírez‐Vélez et al., 2023; Singh et al., 2022; Szekely et al., 2021).

Non‐invasive measurements were performed in the present study. The absence of muscle biopsies did not allow us to compare our patients with those followed by previous authors (Appelman et al., 2024; Colosio et al., 2023) who described, in long COVID patients tested after shorter follow‐ups, dysfunctions of mitochondria and their respiratory function. In this respect, however, it should be acknowledged that the determination of ${\dot{V}}_{O_{2}m}$ kinetics by NIRS in the recovery phase following CWR exercise represents an accepted method of functional evaluation in vivo of skeletal muscle oxidative metabolism (Zuccarelli et al., 2020).

In conclusion, more than 2 years after an initial infection with the SARS‐CoV2 virus, some patients still reported debilitating symptoms such as excessive or premature fatigue, excessive or early dyspnoea on exertion, muscle weakness and myalgias, which negatively affected exercise tolerance and quality of life. In these patients, however, variables evaluating several pathophysiological mechanisms potentially responsible for the symptoms (habitual level of physical activity, muscle size and strength, maximal aerobic power and the ventilatory thresholds, pulmonary ${\dot{V}}_{O_{2}}$ kinetics, peak cardiac function, mechanical efficiency of cycling, microvascular/endothelial function, skeletal muscle oxidative metabolism and mitochondrial respiration evaluated in vivo) were not different compared to what observed in patients with no symptoms. The only exception was represented by evidence of ventilatory inefficiency in a subgroup of the patients with symptoms. This discrepancy between the presence of symptoms and the absence of pathophysiological mechanisms, among those investigated in the present study, was not described in previous studies with shorter follow‐up periods after the original infection. Thus, more than 2 years after the original SARS‐CoV‐2 infection, the potential pathophysiological mechanisms responsible for fatigue and other exercise‐intolerance related symptoms in some long COVID patients are unclear. A complex interaction between physiological and neurological/psychological factors (Kluger et al., 2013; Sherif et al., 2023; Soares et al., 2022; Thomas et al., 2024), or other pathophysiological mechanisms not specifically investigated in the present study may be involved.

## AUTHOR CONTRIBUTIONS

Giovanni Baldassarre, Lucrezia Zuccarelli, Carlo Tascini and Bruno Grassi conceived and designed research. Giovanni Baldassarre, Lucrezia Zuccarelli, Thomas Favaretto, Caterina Ursella and Andrea Palomba performed experiments. Giovanni Baldassarre, Thomas Favaretto, and Andrea Palomba analysed data. Giovanni Baldassarre, Lucrezia Zuccarelli, Carlo Tascini and Bruno Grassi interpreted results of experiments. Giovanni Baldassarre prepared figures. Giovanni Baldassarre and Bruno Grassi drafted manuscript. Giovanni Baldassarre, Lucrezia Zuccarelli, Thomas Favaretto, Caterina Ursella, Andrea Palomba, Paulo Cesar do Nascimento Salvador, Emanuela Sozio, Ernesto Crisafull, Massimo Imazio, Carlo Tascini and Bruno Grassi edited and revised the final version of manuscript. All authors have read and approved the final version of this manuscript and agree to be accountable for all aspects of the work in ensuring that questions related to the accuracy or integrity of any part of the work are appropriately investigated and resolved. All persons designated as authors qualify for authorship, and all those who qualify for authorship are listed.

## CONFLICT OF INTEREST

The authors declare no conflicts of interest.

## Data Availability

The datasets used and analysed during the current study is available from the corresponding author on reasonable request.
